# Beyond words: the association of hugging with loneliness, social isolation, and social withdrawal. Evidence from the general adult population in Germany based on a cross-sectional survey

**DOI:** 10.3389/fpubh.2026.1716316

**Published:** 2026-03-16

**Authors:** André Hajek, Angelina R. Sutin, Martina Luchetti, Yannick Stephan, Karl Peltzer, Supa Pengpid, Dong Keon Yon, Razak M. Gyasi, Andrew Stickley, Antonio Terracciano, Hans-Helmut König

**Affiliations:** 1 Department of Health Economics and Health Services Research, University Medical Center Hamburg-Eppendorf, Hamburg Center for Health Economics, Hamburg, Germany; 2 Department of Behavioral Sciences and Social Medicine, Florida State University College of Medicine, Tallahassee, FL, United States; 3 Euromov, University of Montpellier, Montpellier, France; 4 Department of Health Education and Behavioral Sciences, Faculty of Public Health, Mahidol University, Bangkok, Thailand; 5 Department of Psychology, University of the Free State, Bloemfontein, South Africa; 6 Department of Medical Research, China Medical University Hospital, China Medical University, Taichung, Taiwan; 7 Department of Public Health, Sefako Makgatho Health Sciences University, Pretoria, South Africa; 8 Department of Healthcare Administration, College of Medical and Health Science, Asia University, Taichung, Taiwan; 9 Center for Digital Health, Medical Science Research Institute, Kyung Hee University College of Medicine, Seoul, Republic of Korea; 10 African Population and Health Research Center, Nairobi, Kenya; 11 National Centre for Naturopathic Medicine, Faculty of Health, Southern Cross University, Lismore, NSW, Australia; 12 Department of Preventive Intervention for Psychiatric Disorders, National Institute of Mental Health, National Center of Neurology and Psychiatry, Tokyo, Japan; 13 Department of Geriatrics, Florida State University College of Medicine, Tallahassee, FL, United States

**Keywords:** embracing, hikikomori, hug, hugging, loneliness, social exclusion, social isolation, social withdrawal

## Abstract

**Background:**

Thus far, no studies have focused on hugging and social disconnectedness outcomes comprehensively (loneliness, social isolation, social withdrawal) in the post-pandemic era. Our aim was to examine whether hugging is associated with loneliness, social isolation, and social withdrawal and whether these associations vary by gender.

**Methods:**

We used cross-sectional data collected via an online survey conducted in January 2025 (N = 3,270 adults aged 18 to 74 years across Germany). To ensure representativeness, a quota sampling method was employed based on gender, age, and federal state. Participants reported on their loneliness, social isolation, and social withdrawal and how many individuals they hugged each day. Linear regression models with robust standard errors were used.

**Results:**

After adjusting for multiple covariates, hugging on average one (or more) individuals per day (compared to lack of hugging) was associated with fewer feelings of loneliness, social isolation, and social withdrawal in the total sample. Among men, hugging was associated with reduced feelings of loneliness and social withdrawal but not social isolation; hugging was related to all three social disconnectedness metrics among women and the associations were stronger than for men, as indicated by corresponding interaction terms (hugging x gender).

**Conclusion:**

Our findings suggested an association between hugging frequency and measures of social disconnectedness, particularly among women. Efforts to increase the frequency of hugs, ideally to at least 2–3 individuals on average per day, may be beneficial for social outcomes, pending longitudinal and experimental evidence.

## 1 Introduction

Loneliness is a negative emotion that refers to the perceived difference between actual and desired social relationships (1), perceived social isolation refers to a feeling of not belonging to the society (2), while and social withdrawal refers to retreating from society (e.g., in terms of one’s relationships and social interactions) (3). These aspects of social disconnection are prevalent across the lifespan, and not limited to old age, and contribute to morbidity and mortality (4, 5) and other unfavorable outcomes (6).

There are various factors are associated with these components of social disconnectedness. Spousal loss, for example, is associated with worse social disconnectedness outcomes (7, 8). Physical and emotional closeness may be important for loneliness, social isolation and social withdrawal (9). Hugs (act of embracing another person, often as a sign of comfort, affection or greeting) can be one such an expression of physical and emotional closeness (10). When individuals are sad, for example, after the end of a relationship, a hug may make them feel less lonely. A daily hug may also intensify relationships as a sign of affection. Similarly, hugs as a sign of greeting may symbolize closeness and a certain depth and quality of friendship.

One previous study that used data from a representative sample of American adults aged 18 to 94 years found that hugging or kissing a romantic partner was significantly associated with less loneliness, whereas hugging or kissing a family member was not significantly associated with loneliness (11). A more recent qualitative study also showed that the lack of hugs during the pandemic was burdensome for low-income adolescents in São Paulo, Brazil (12). However, no studies have focused specifically on *hugging* and *social disconnectedness outcomes more comprehensively* (loneliness, social isolation, social withdrawal) in the *post-pandemic era*. Therefore, we aimed to examine the association between hugging and loneliness, social isolation, and social withdrawal and whether these associations vary by gender. Since the meaning and importance of hugs may vary depending on gender (with potentially stronger associations among women) (13, 14), the role of gender in the association between hugging and social disconnectedness outcomes will be explored. Such knowledge is of great importance to better characterize individuals at risk of high levels of loneliness, social isolation and social withdrawal. This in turn is important as such social disconnectedness outcomes can eventually lead to chronic conditions and premature death (4, 5).

## 2 Methods

### 2.1 Sample

Data were drawn from an online survey that was conducted in January 2025, involving 3,270 participants aged 18 to 74 years who were residing in Germany. The only inclusion criteria were being within the specified age range and living in Germany. The survey was administered by Bilendi (a reputable ISO-certified market research company). To ensure that the sample accurately reflected the German adult population (in terms of gender, age, and federal state) a quota sampling strategy was used.

### 2.2 Dependent variables

Loneliness was assessed using the 6-item De Jong Gierveld (DJG) Loneliness Scale (15). Items were averaged. The average score could range from 0 to 6; higher scores indicated higher loneliness levels (Cronbach’s alpha = 0.77; McDonald’s omega = 0.75).

Social isolation was measured using the 4-item Bude and Lantermann tool (2). Responses were averaged to create a final score that ranged from 1 to 4, higher scores indicated higher levels of social isolation (Cronbach’s alpha and McDonald’s omega both = 0.91).

Social withdrawal was measured with the validated German version (16) of the 25-item Hikikomori Questionnaire (HQ-25) (17). Items were summed, and scores ranged from 0 to 100, higher scores indicated more social withdrawal (Cronbach’s alpha and McDonald’s omega both = 0.92).

### 2.3 Main independent variable: frequency of hugs

Participants reported the number of individuals they hugged on average on a day. Following former research (18), individuals could indicate a frequency ranging from 0 to 99 individuals. Based on this and similar to previous research (18), four categories were developed: The first category included individuals who answered 0 (i.e., on average, they did not hug anyone per day). The second category included individuals who hugged one individual per day on average. The third category included people who hugged two or three individuals per day on average. The fourth category included individuals who hugged four or more individuals per day on average.

### 2.4 Covariates

Covariates for regression analysis were selected based on prior research (19, 20) and theoretical grounds. For example, lifestyle factors may affect the likelihood of hugs (e.g., before or after joint sporting activities). Physical activity can also have a direct effect on outcomes such as loneliness (21). Moreover, health-related factors such as chronic illnesses may affect the ability to maintain social contacts (e.g., receiving hugs) and the quality of relationships (which can affect social outcomes such as loneliness) (22).

Sociodemographic covariates were age, gender (men, women, diverse), marital status (single, divorced, widowed, cohabiting—either married/in a partnership, living separately, but married/in a partnership), size of the city (less than 2,000 inhabitants, 2,000 to less than 5,000 inhabitants, 5,000 to less than 20,000 inhabitants, 20,000 to less than 50,000 inhabitants, 50,000 to less than 100,000 inhabitants, 100,000 to less than 500,000 inhabitants, 500,000 inhabitants and more), education (according to the ISCED-97 framework (23): low, medium, or high), employment status (full-time employed, retired, or other), and religious affiliation (no religious affiliation, Christianity, Islam, other). Lifestyle factors were frequency of physical activity (from no activity to more than 4 h weekly; five categories in total), alcohol intake (daily, several times a week, once a week, 1–3 times monthly, less often, or never), and smoking history (never smoked, former smoker, occasional smoker, or daily smoker).

Health-related factors included self-rated health (single item, from 1 = very poor to 5 = very good), sum of chronic conditions (based on 15 chronic conditions such as cancer or diabetes), and probable depression [Patient Health Questionnaire-9 (PHQ-9) ≥ 10 (24)].

### 2.5 Statistical analysis

Descriptive statistics were computed to evaluate the characteristics of the sample (also stratified by the frequency of hugs). Unadjusted and adjusted linear regression analyses were calculated with robust standard errors. In the adjusted models, initial covariates were sociodemographic factors. Lifestyle-related factors were then incorporated as additional covariates, followed by health-related covariates. Moreover, moderation by gender was tested (by including interaction terms for frequency of hugging x gender).

Effect sizes were also calculated (Cohen’s *d* for unadjusted comparisons and partial Eta^2^ for regression analysis). Cohen’s *d* = 0.20 reflects a small effect, *d* = 0.50 reflects a medium effect and *d* = 0.80 reflects a large effect size (25). Partial eta-squared values can be classified as follows (25): 0.01 as “small,” 0.06 as “medium, and 0.14 as “large”, respectively. McDonald’s omega was calculated using the “omegacoef” command in Stata to estimate internal consistency for the scales (26).

The threshold for statistical significance was set at *p* < 0.05. All analyses were conducted using Stata Now 18.5 MP-Parallel Edition (StataCorp, College Station, Texas).

## 3 Results

### 3.1 Sample characteristics

Characteristics of the sample are in Table 1 (*n* = 3,270 individuals). Overall, 50.4% of the respondents were women and the average age was 47.0 years (SD: 15.3 years, range 18–74 years).

**Table 1:** Sample characteristics stratified by frequency of hugs (*n* = 3,270).

Variables	Average number of individuals hugged per day				Total	*p*-value
	0	1	2 or 3	4 or more		
In total	814 (24.9)	1,251 (38.3)	998 (30.5)	207 (6.3)	3,270 (100.0)	
Gender: *N* (%)						0.24
Men	434 (53.3)	605 (48.4)	477 (47.8)	98 (47.3)	1,614 (49.4)	
Women	377 (46.3)	643 (51.4)	518 (51.9)	109 (52.7)	1,647 (50.4)	
Diverse	3 (0.4)	3 (0.2)	3 (0.3)	0 (0.0)	9 (0.3)	
Age: mean (SD)	49.6 (15.2)	47.6 (15.9)	44.6 (14.3)	43.7 (13.7)	47.0 (15.3)	<0.001
Marital situation: *N* (%)						<0.001
Single	402 (49.4)	308 (24.6)	160 (16.0)	38 (18.4)	908 (27.8)	
Divorced	133 (16.3)	77 (6.2)	52 (5.2)	15 (7.2)	277 (8.5)	
Widowed	56 (6.9)	27 (2.2)	17 (1.7)	3 (1.4)	103 (3.1)	
Married, cohabiting with spouse	192 (23.6)	790 (63.1)	737 (73.8)	148 (71.5)	1867 (57.1)	
Married, not cohabiting with spouse	31 (3.8)	49 (3.9)	32 (3.2)	3 (1.4)	115 (3.5)	
Educational level: *N* (%)						<0.001
Low	114 (14.0)	94 (7.5)	107 (10.7)	22 (10.6)	337 (10.3)	
Medium	412 (50.6)	627 (50.1)	422 (42.3)	91 (44.0)	1,552 (47.5)	
High	288 (35.4)	530 (42.4)	469 (47.0)	94 (45.4)	1,381 (42.2)	
Labor force participation: *N* (%)						<0.001
Full-time employed	360 (44.2)	611 (48.8)	540 (54.1)	118 (57.0)	1,629 (49.8)	
Retired	209 (25.7)	292 (23.3)	129 (12.9)	19 (9.2)	649 (19.8)	
Other	245 (30.1)	348 (27.8)	329 (33.0)	70 (33.8)	992 (30.3)	
City size: *N* (%)						0.11
Less than 2,000 inhabitants	50 (6.1)	86 (6.9)	61 (6.1)	13 (6.3)	210 (6.4)	
2,000 to less than 5,000 inhabitants	66 (8.1)	85 (6.8)	69 (6.9)	13 (6.3)	233 (7.1)	
5,000 to less than 20,000 inhabitants	197 (24.2)	270 (21.6)	210 (21.0)	47 (22.7)	724 (22.1)	
20,000 to less than 50,000 inhabitants	132 (16.2)	217 (17.3)	184 (18.4)	17 (8.2)	550 (16.8)	
50,000 to less than 100,000 inhabitants	89 (10.9)	114 (9.1)	101 (10.1)	23 (11.1)	327 (10.0)	
100,000 to less than 500,000 inhabitants	124 (15.2)	204 (16.3)	177 (17.7)	40 (19.3)	545 (16.7)	
500,000 inhabitants and more	156 (19.2)	275 (22.0)	196 (19.6)	54 (26.1)	681 (20.8)	
Religious affiliation: *N* (%)						<0.001
No religious affiliation	392 (48.2)	628 (50.2)	365 (36.6)	74 (35.7)	1,459 (44.6)	
Christianity	388 (47.7)	578 (46.2)	582 (58.3)	111 (53.6)	1,659 (50.7)	
Islam	21 (2.6)	24 (1.9)	31 (3.1)	14 (6.8)	90 (2.8)	
Other	13 (1.6)	21 (1.7)	20 (2.0)	8 (3.9)	62 (1.9)	
Smoking status: *N* (%)						<0.001
Yes, daily	221 (27.1)	246 (19.7)	219 (21.9)	51 (24.6)	737 (22.5)	
Yes, sometimes	49 (6.0)	117 (9.4)	140 (14.0)	41 (19.8)	347 (10.6)	
No, not anymore	203 (24.9)	378 (30.2)	255 (25.6)	55 (26.6)	891 (27.2)	
Never smoker	341 (41.9)	510 (40.8)	384 (38.5)	60 (29.0)	1,295 (39.6)	
Alcohol consumption: *N* (%)						<0.001
Daily	54 (6.6)	57 (4.6)	58 (5.8)	16 (7.7)	185 (5.7)	
Several times a week	116 (14.3)	205 (16.4)	193 (19.3)	46 (22.2)	560 (17.1)	
Once a week	110 (13.5)	244 (19.5)	191 (19.1)	33 (15.9)	578 (17.7)	
1–3 times a month	121 (14.9)	230 (18.4)	181 (18.1)	26 (12.6)	558 (17.1)	
Less often	212 (26.0)	315 (25.2)	211 (21.1)	44 (21.3)	782 (23.9)	
Never	201 (24.7)	200 (16.0)	164 (16.4)	42 (20.3)	607 (18.6)	
Sports activity: *N* (%)						<0.001
No sports activity	322 (39.6)	312 (24.9)	181 (18.1)	28 (13.5)	843 (25.8)	
Less than 1 h a week	124 (15.2)	259 (20.7)	187 (18.7)	35 (16.9)	605 (18.5)	
Regularly, 1–2 h a week	160 (19.7)	309 (24.7)	287 (28.8)	57 (27.5)	813 (24.9)	
Regularly, 2–4 h a week	95 (11.7)	210 (16.8)	204 (20.4)	41 (19.8)	550 (16.8)	
Regularly, more than 4 h a week	113 (13.9)	161 (12.9)	139 (13.9)	46 (22.2)	459 (14.0)	
Probable depression (PHQ-9): *N* (%)						<0.01
No	541 (66.5)	915 (73.1)	739 (74.0)	148 (71.5)	2,343 (71.7)	
Yes	273 (33.5)	336 (26.9)	259 (26.0)	59 (28.5)	927 (28.3)	0.00
Count of chronic conditions: mean (SD)	2.0 (2.0)	1.7 (1.7)	1.5 (1.7)	1.6 (1.7)	1.7 (1.8)	<0.001
Self-rated health: mean (SD)	3.3 (0.9)	3.5 (0.8)	3.7 (0.8)	3.8 (0.8)	3.6 (0.8)	<0.001
Loneliness (DJG-6): mean (SD)	4.1 (1.9)	3.2 (2.0)	2.9 (2.0)	2.8 (2.0)	3.3 (2.0)	<0.001
Social isolation (Bude and Lantermann tool): mean (SD)	2.1 (0.9)	1.9 (0.8)	1.8 (0.8)	1.9 (0.8)	2.0 (0.8)	<0.001
Social withdrawal (HQ-25-G): mean (SD)	46.9 (17.0)	37.4 (16.0)	32.6 (16.3)	33.3 (17.5)	38.0 (17.3)	<0.001

*p*-values are based on one-way ANOVAs or chi-square tests, as appropriate.

In total, 24.9% of respondents reported that they did not hug anyone on average daily, 38.3% of respondents reported that they hugged 1 individual per day, 30.5% of respondents reported that they hugged 2 to 3 individuals on average per day, and 6.3% of respondents reported that they hugged 4 or more individuals on average per day.

Depending on frequency of hugs, the mean loneliness score varied from 2.8 (SD: 2.0; among individuals hugging 4 or more individuals on average each day) to 4.1 (SD: 1.9; among individuals hugging nobody on average each day). This difference corresponded to an effect size Cohen’s *d* = 0.7. Mean social isolation varied from 1.8 (SD: 0.8; among individuals hugging two or three individuals on average daily) to 2.1 (SD: 0.9; among individuals hugging nobody on average daily). This difference corresponded to an effect size Cohen’s *d* = 0.4. Mean social withdrawal varied from 32.6 (SD: 16.3; among individuals hugging two or three individuals on average daily) to 46.9 (SD: 17.0; among individuals hugging nobody on average daily). This difference corresponded to an effect size Cohen’s *d* = 0.9. Additional details are in Table 1.

### 3.2 Regression analysis

The results of the unadjusted and adjusted linear regressions (examining the association between the frequency of hugs and loneliness, social isolation and social withdrawal) are in Table 2. Gender-stratified regressions are in Table 3 (men) and Table 4 (women). The fully-adjusted models indicated that hugging on average one or more individuals per day (compared to the lack of hugging) was associated with less loneliness, social isolation, and social withdrawal in the total sample (see Table 2). The largest difference in terms of loneliness was between individuals who hug on average nobody daily, and people who hug at least four people daily on average (*β* = −1.03, *p* < 0.001). The largest differences for social isolation and social withdrawal were between individuals who hug two to three individuals on average every day and those who do not hug anyone (*β* = −0.20, *p* < 0.001 and *β* = −10.54, *p* < 0.001, respectively).

**Table 2:** Association of hugging frequency with loneliness, social isolation, and social withdrawal among the total sample based on linear regressions.

Independent variables	Loneliness				Perceived social isolation				Social withdrawal			
	Model 1	Model 2	Model 3	Model 4	Model 1	Model 2	Model 3	Model 4	Model 1	Model 2	Model 3	Model 4
Hugging on average one individual per day (compared to the lack of hugging)	−0.86***	−0.71***	−0.72***	−0.59***	−0.17***	−0.16***	−0.17***	−0.10**	−9.44***	−8.18***	−7.54***	−6.45***
	(−1.03–−0.69)	(−0.89–−0.53)	(−0.90–−0.54)	(−0.76–−0.42)	(−0.25–−0.10)	(−0.24–−0.08)	(−0.25–−0.09)	(−0.17–−0.03)	(−10.91–−7.98)	(−9.71–−6.65)	(−9.05–−6.03)	(−7.87–−5.03)
Hugging on average two or three individuals per day	−1.16***	−1.05***	−1.08***	−0.85***	−0.28***	−0.30***	−0.31***	−0.20***	−14.30***	−13.11***	−12.40***	−10.54***
	(−1.34–−0.99)	(−1.24–−0.86)	(−1.27–−0.88)	(−1.04–−0.66)	(−0.36–−0.20)	(−0.38–−0.22)	(−0.40–−0.23)	(−0.28–−0.13)	(−15.84–−12.75)	(−14.76–−11.46)	(−14.04–−10.76)	(−12.11–−8.98)
Hugging on average four or more individuals per day	−1.26***	−1.20***	−1.26***	−1.03***	−0.19**	−0.22***	−0.24***	−0.14*	−13.61***	−12.72***	−12.03***	−10.23***
	(−1.56–−0.96)	(−1.51–−0.90)	(−1.56–−0.96)	(−1.32–−0.74)	(−0.32–−0.06)	(−0.34–−0.10)	(−0.36–−0.12)	(−0.25–−0.02)	(−16.26–−10.96)	(−15.33–−10.10)	(−14.59–−9.47)	(−12.70–−7.75)
Sociodemographic covariates		✓	✓	✓		✓	✓	✓		✓	✓	✓
Lifestyle-related covariates			✓	✓			✓	✓			✓	✓
Health-related covariates				✓				✓				✓
*R* ^2^	3,270	3,270	3,270	3,270	3,270	3,270	3,270	3,270	3,270	3,270	3,270	3,270
Observations	0.05	0.10	0.12	0.23	0.02	0.12	0.13	0.30	0.10	0.17	0.20	0.31

*** *p* < 0.001, ** *p* < 0.01, * *p* < 0.05, + *p* < 0.10, unstandardized beta-coefficients are shown (95% CI are displayed in parentheses); sociodemographic factors encompass gender, age, size of the city, education, marital situation, labor force participation, and religious affiliation; lifestyle-related factors encompass smoking status, sports activities and alcohol consumption; health-related factors encompass probable depression, the number of chronic illnesses, and self-rated health. Model 1 is without covariates, model 2 includes sociodemographic covariates, model 3 includes sociodemographic and lifestyle-related covariates, and model 4 includes sociodemographic, lifestyle-related, and health-related covariates.

**Table 3:** Association of hugging frequency with loneliness, social isolation, and social withdrawal among men based on linear regressions.

Independent variables	Loneliness				Perceived social isolation				Social withdrawal			
	Model 1	Model 2	Model 3	Model 4	Model 1	Model 2	Model 3	Model 4	Model 1	Model 2	Model 3	Model 4
Hugging on average one individual per day (compared to the lack of hugging)	−0.68***	−0.43***	−0.45***	−0.36**	−0.10+	−0.03	−0.04	0.01	−7.68***	−5.79***	−5.18***	−4.43***
	(−0.91–−0.45)	(−0.67–−0.18)	(−0.70–−0.20)	(−0.60–−0.13)	(−0.20–0.01)	(−0.14–0.08)	(−0.15–0.07)	(−0.09–0.10)	(−9.72–−5.64)	(−7.96–−3.62)	(−7.34–−3.02)	(−6.41–−2.44)
Hugging on average two or three individuals per day	−0.96***	−0.73***	−0.74***	−0.55***	−0.16**	−0.13*	−0.15*	−0.05	−12.54***	−10.71***	−10.02***	−8.42***
	(−1.21–−0.71)	(−1.00–−0.46)	(−1.02–−0.47)	(−0.82–−0.29)	(−0.27–−0.05)	(−0.24–−0.01)	(−0.26–−0.03)	(−0.15–−0.06)	(−14.73–−10.34)	(−13.09–−8.33)	(−12.38–−7.65)	(−10.66–−6.19)
Hugging on average four or more individuals per day	−0.82***	−0.70**	−0.76***	−0.64**	0.03	0.03	−0.00	0.05	−10.24***	−8.99***	−8.71***	−7.93***
	(−1.26–−0.38)	(−1.16–−0.25)	(−1.21–−0.31)	(−1.05–−0.22)	(−0.14–0.21)	(−0.15–−0.20)	(−0.18–−0.18)	(−0.12–−0.22)	(−14.16–−6.32)	(−12.91–−5.07)	(−12.52–−4.91)	(−11.47–−4.39)
Sociodemographic covariates		✓	✓	✓		✓	✓	✓		✓	✓	✓
Lifestyle-related covariates			✓	✓			✓	✓			✓	✓
Health-related covariates				✓				✓				✓
*R* ^2^	1,614	1,614	1,614	1,614	1,614	1,614	1,614	1,614	1,614	1,614	1,614	1,614
Observations	0.04	0.09	0.10	0.21	0.01	0.11	0.13	0.33	0.08	0.15	0.19	0.32

*** *p* < 0.001, ** *p* < 0.01, * *p* < 0.05, + *p* < 0.10, unstandardized beta-coefficients are shown (95% CI are displayed in parentheses); sociodemographic factors encompass age, size of the city, education, marital situation, labor force participation, and religious affiliation; lifestyle-related factors encompass smoking status, sports activities and alcohol consumption; health-related factors encompass probable depression, the number of chronic illnesses, and self-rated health. Model 1 is without covariates, model 2 includes sociodemographic covariates, model 3 includes sociodemographic and lifestyle-related covariates, and model 4 includes sociodemographic, lifestyle-related, and health-related covariates.

**Table 4:** Association of hugging frequency with loneliness, social isolation and social withdrawal among women based on linear regressions.

Independent variables	Loneliness				Perceived social isolation				Social withdrawal			
	Model 1	Model 2	Model 3	Model 4	Model 1	Model 2	Model 3	Model 4	Model 1	Model 2	Model 3	Model 4
Hugging on average one individual per day (compared to the lack of hugging)	−1.04***	−0.99***	−0.97***	−0.81***	−0.26***	−0.30***	−0.30***	−0.22***	−11.23***	−10.82***	−10.05***	−8.78***
	(−1.29–-0.79)	(−1.25–-0.72)	(−1.24–-0.71)	(−1.06–-0.55)	(−0.37–-0.16)	(−0.41–-0.19)	(−0.41–-0.19)	(−0.32–-0.12)	(−13.35–-9.11)	(−13.01–-8.62)	(−12.21–-7.88)	(−10.84–-6.73)
Hugging on average two or three individuals per day	−1.38***	−1.38***	−1.40***	−1.13***	−0.41***	−0.48***	−0.48***	−0.35***	−16.08***	−15.82***	−14.98***	−13.00***
	(−1.63–-1.12)	(−1.65–-1.10)	(−1.67–-1.12)	(−1.40–-0.86)	(−0.52–-0.30)	(−0.59–-0.36)	(−0.59–-0.36)	(−0.46–-0.25)	(−18.28–-13.88)	(−18.14–-13.50)	(−17.29–-12.68)	(−15.21–-10.78)
Hugging on average four or more individuals per day	−1.68***	−1.70***	−1.73***	−1.44***	−0.41***	−0.47***	−0.48***	−0.34***	−16.77***	−16.75***	−15.59***	−13.30***
	(−2.09–-1.27)	(−2.11–-1.29)	(−2.13–-1.33)	(−1.84–-1.04)	(−0.59–-0.23)	(−0.64–-0.30)	(−0.65–-0.31)	(−0.50–-0.18)	(−20.32–-13.21)	(−20.21–-13.28)	(−19.04–-12.14)	(−16.76–-9.83)
Sociodemographic covariates		✓	✓	✓		✓	✓	✓		✓	✓	✓
Lifestyle-related covariates			✓	✓			✓	✓			✓	✓
Health-related covariates				✓				✓				✓
*R* ^2^	1,647	1,647	1,647	1,647	1,647	1,647	1,647	1,647	1,647	1,647	1,647	1,647
Observations	0.07	0.12	0.15	0.25	0.03	0.14	0.16	0.31	0.13	0.20	0.23	0.31

*** *p* < 0.001, ** *p* < 0.01, * *p* < 0.05, + *p* < 0.10, unstandardized beta-coefficients are shown (95% CI are displayed in parentheses); Model 1 sociodemographic factors encompass age, size of the city, education, marital situation, labor force participation, and religious affiliation; lifestyle-related factors encompass smoking status, sports activities and alcohol consumption; health-related factors encompass probable depression, the number of chronic illnesses, and self-rated health. Model 1 is without covariates, model 2 includes sociodemographic covariates, model 3 includes sociodemographic and lifestyle-related covariates, and model 4 includes sociodemographic, lifestyle-related, and health-related covariates.

Among men, hugging on average one or more individuals per day (compared to the lack of hugging) was associated with less loneliness and social withdrawal (see Table 3). Significant differences between those who hug two to three individuals on average daily and those who do not hug anyone were reduced to non-significance adjusting for health-related covariates among men.

Among women, hugging on average one or more individuals per day (compared to the lack of hugging) was associated with less loneliness, social isolation and social withdrawal (see Table 4). It is worth noting that *all* associations between the frequency of hugging and the three outcomes were significantly more pronounced among women, as indicated by significant interaction terms for frequency of hugging x gender. Please see Table 5 for further details.

**Table 5:** Association of hugging frequency with loneliness, social isolation and social withdrawal among the total sample based on linear regressions (with interaction term: hugging x gender).

Independent variables	Loneliness	Social isolation	Social withdrawal
Hugging on average one individual per day (compared to the lack of hugging)	−0.42***	−0.02	−4.69***
	(−0.64–−0.19)	(−0.11–0.08)	(−6.57–−2.81)
Hugging on average two or three individuals per day	−0.63***	−0.08	−8.75***
	(−0.88–−0.39)	(−0.18–0.02)	(−10.84–−6.67)
Hugging on average four or more individuals per day	−0.70***	0.02	−7.86***
	(−1.10–−0.29)	(−0.14–0.18)	(−11.29–−4.42)
Gender: women (compared to men)	0.16	0.14**	−0.02
	(−0.09–0.41)	(0.04–0.25)	(−2.16–2.12)
Interaction term (hugging x gender): Hugging on average one individual per day x women	−0.37*	−0.19**	−3.77**
	(−0.69–−0.05)	(−0.31–−0.06)	(−6.40–−1.13)
Hugging on average two or three individuals per day x women	−0.46**	−0.25***	−3.75**
	(−0.79–−0.12)	(−0.38–−0.11)	(−6.54–−0.97)
Hugging on average four or more individuals per day x women	−0.69*	−0.34**	−4.84*
	(−1.24–−0.15)	(−0.55–−0.12)	(−9.52–−0.17)
Sociodemographic covariates	✓	✓	✓
Lifestyle-related covariates	✓	✓	✓
Health-related covariates	✓	✓	✓
*R* ^2^	3,261	3,261	3,261
Observations	0.23	0.31	0.31

*** *p* < 0.001, ** *p* < 0.01, * *p* < 0.05, + *p* < 0.10, unstandardized beta-coefficients are shown (95% CI are displayed in parentheses); sociodemographic factors encompass gender, age, size of the city, education, marital situation, labor force participation, and religious affiliation; lifestyle-related factors encompass smoking status, sports activities and alcohol consumption; health-related factors encompass probable depression, the number of chronic illnesses, and self-rated health. The model includes sociodemographic, lifestyle-related, and health-related covariates.

Among the total sample, the effect sizes (in terms of partial eta-squared) for the frequency of hugging were 0.03 for loneliness, 0.01 for social isolation, and 0.06 for social withdrawal, corresponding to small to medium effect sizes. Among men, the effect sizes for frequency of hugging were 0.01 for loneliness, 0.001 for social isolation, and 0.04 for social withdrawal, reflecting small effect sizes. Among women, the effect sizes for frequency of hugging were 0.05 for loneliness, 0.03 for social isolation, and 0.08 for social withdrawal, corresponding to small to medium effect sizes.

## 4 Discussion

Our objective was to investigate whether hugging was associated with loneliness, social isolation, and social withdrawal and whether these associations varied by gender. Our main findings were that hugging on average one or more individuals per day (compared to no hugging) was associated with feeling less loneliness, social isolation, and social withdrawal among the total sample. These associations were more pronounced among women. The associations were weaker and still apparent for loneliness and social withdrawal among men.

As there has been little research to date on the association of hugging with loneliness, social isolation and social withdrawal it is challenging to contextualize our findings in terms of previous research. As outlined in the introduction, one former study (11) indicated that hugging or kissing a romantic partner was associated with lower loneliness among American adults in the early stages of the Covid-19 pandemic. Our current study builds on this previous Covid-19 specific American study (11) and shows that the frequency of hugging is associated with loneliness and also social isolation and social withdrawal in Germany in the post-pandemic era.

The frequency of *daily* hugging may be particularly driven by hugs as a sign of greeting or affection rather than comfort. In this respect, the physical closeness of a hug may make individuals aware of their favorable and high-quality current relationships (e.g., to friends and acquaintances), ultimately contributing to lower levels of loneliness, social isolation and social withdrawal. Even if sad, being hugged might make individuals feel connected and included in society (12). Interestingly, being hugged on average two or three times daily seems to be sufficient (in terms of the outcomes used in this study) (27). This frequency of hugging may reflect a mix of adequate (in terms of number of social contacts) yet high-quality social relationships (11). Future research is needed to investigate this in more detail.

In accordance with our initial assumptions, the associations of interest were significantly more pronounced among women than men. This difference may be explained by the fact that hugs might be more relevant for women as an expression of physical closeness in their friendships (or other relationships) (14). Hugs may be more important to express their emotions and receive support (14). Emotional closeness (also expressed by hugs) may be particularly important for women to feel connected in their social relationships. Cultural norms and social expectations may also be of relevance. More precisely, we assume that hugging as a sign of physical closeness may be more common among women friends in Germany than among men friends. If women then hug less, for example, they could quickly feel lonely, isolated and withdrawn because their expectations are not met. Biologically, physical touches such as hugs may promote the release of oxytocin (a hormone related to stress reduction, trust, and bonding) (28, 29). For reasons of biological responses (30) and social customs, women may particularly benefit from such effects.

When interpreting our present findings, it is important to consider some key strengths and limitations of this work. The data were from a large sample that reflects the German adult population – in terms of gender, age groups, and federal states. Psychometrically sound tools were used to quantify the outcomes. However, solely subjective tools were used. Future research could focus on, for example, objective factors to quantify social withdrawal. While the tool used to quantify hugging has a high face validity, more sophisticated tools are recommended. For example, future research could distinguish between different types of hugs, such as hugs between relatives, friends or partners. It would also be interesting to examine the duration of hugs in more detail. Furthermore, the context and intimacy of hugs should be explored in future research. The observational study design has shortcomings regarding the directionality and causality of the relationship (e.g., individuals who withdraw from society may be also less likely to hug others). It is possible that people who are feeling lonely and isolated are too distressed to socialize, or have fewer people to hug, or they may even prefer greater social distance. Even if social disconnection leads to fewer hugs, it is still possible that an intervention based on hugs could reduce loneliness and social disconnection. Therefore, experimental longitudinal studies are recommended to get a better understanding of the directionality and evaluate causality. Additionally, a potential online response bias cannot be completely excluded. Nonetheless, we believe that responding to an online survey may lead to more honest answers (e.g., in terms of the actual number of hugs per day) compared to being interviewed in person (31). Another limitation is that it was not possible to adjust for social network size. Such a factor could be included in future research.

In conclusion, our findings suggested an association between hugging frequency and measures of social disconnectedness – among women in particular. Efforts to increase the frequency of hugs, ideally to at least two to three individuals on average daily may be beneficial for such social disconnectedness outcomes, pending longitudinal and experimental evidence. Since the frequency and social meaning of hugging may vary between countries (e.g., Western and Asian cultures), future cross-country comparisons may be of interest. Some individuals experienced post-pandemic stress disorder due to the COVID-19 pandemic, with resulting behavioral changes. Such a topic could also be further explored in future studies in this context by explicitly focusing on this subgroup.

## Data Availability

The datasets presented in this article are not readily available because the data are not publicly available due to ethical restrictions. Requests to access the datasets should be directed to the corresponding author and the Department for Health Economics and Health Services Research, University Medical Center Hamburg-Eppendorf: igv@uke.de.
